# Genomic variations and signatures of selection in Wuhua yellow chicken

**DOI:** 10.1371/journal.pone.0241137

**Published:** 2020-10-23

**Authors:** Zhuoxian Weng, Yongjie Xu, Weina Li, Jiebo Chen, Ming Zhong, Fusheng Zhong, Bingwang Du, Bin Zhang, Xunhe Huang

**Affiliations:** 1 College of Animal Science and Technology, Hunan Agricultural University, Changsha, China; 2 Guangdong Provincial Key Laboratory of Conservation and Precision Utilization of Characteristic Agricultural Resources in Mountainous Areas, Meizhou, China; 3 Guangdong Innovation Centre for Science and Technology of Wuhua Yellow Chicken, Meizhou, China; 4 School of Life Science, Jiaying University, Meizhou, China; UCSI University, MALAYSIA

## Abstract

Wuhua yellow chicken (WHYC) is an important traditional yellow-feathered chicken from China, which is characterized by its white tail feathers, white flight feathers, and strong disease resistance. However, the genomic basis of these unique traits associated with WHYC is poorly understood. In this study, whole-genome resequencing was performed with an average coverage of 20.77-fold to investigate heritable variation and identify selection signals in WHYC. Reads were mapped onto the chicken reference genome (Galgal5) with a coverage of 85.95%. After quality control, 11,953,471 single nucleotide polymorphisms and 1,069,574 insertion/deletions were obtained. In addition, 41,408 structural variants and 33,278 copy number variants were found. Comparative genomic analysis of WHYC and other yellow-feathered chicken breeds showed that selected regions were enriched in genes involved in transport and catabolism, immune system, infectious diseases, signal transduction, and signaling molecules and interactions. Several genes associated with disease resistance were also identified, including *IFNA*, *IFNB*, *CD86*, *IL18*, *IL11RA*, *VEGFC*, and *ATG10*. Furthermore, our results suggest that *PMEL* and *TYRP1* may contribute to the white feather coloring in WHYC. These findings can improve our understanding of the genetic characteristics of WHYC and may contribute to future breed improvement.

## 1. Introduction

Wuhua yellow chicken (WHYC) is a unique breed that is mainly found in Meizhou city of Guangdong Province, China as a native small-type broiler chicken. WHYC is a breed of traditional yellow-feathered chickens (YFCs) identifiable by its white tail feathers and white flight feathers in addition to other desirable characteristics such as good meat quality and strong disease resistance [1, 2]. In the 1970s and 1980s, Hong Kong, Macao, and Southeast Asia were the major markets for WHYCs; however, the rapid expansion of commercial chickens has led to a dramatic decrease in the number of WHYCs [3]. Currently, roughly 3000 live chickens of the WHYC breed exist, which are mainly kept at a breeding farm and in some remote mountainous areas. As a small indigenous population, WHYCs are at risk of extinction; thus, protection of this genetic resource is urgently required.

Previous studies of WHYC have primarily focused on breed characters and population genetics. According to Zhong et al. [1, 4], WHYC has a high slaughter rate and good meat quality. The high protein and low-fat contents of its meat are in line with the current concept of a “healthy diet” [5]. Similar to other Chinese indigenous chickens, WHYC has the disadvantages of a slow growth rate and low reproductive performance [1, 6]. WHYC may have originated in Southeast Asia and its evolution was likely influenced by indigenous chickens in neighboring provinces [7]. Genetic marker analyses such as analyses of mitochondrial DNA and microsatellites have uncovered the genetic features and population structure of WHYCs, demonstrating high genetic diversity [7–9]. Additional research has focused on purification and rejuvenation, breeding conservation and selection, ecological farming, and product processing [5, 10, 11]. However, systematic studies of the molecular mechanisms underlying disease resistance and its unique feather color characteristic, as well as the genomic basis of the breeding history and economic traits of WHYC are lacking. This paucity of information is not conducive to rational improvement and conservation.

With the aim of enriching the genetic background and evaluating the unique characteristics of WHYC, we performed whole-genome sequencing of 12 WHYCs and conducted a comparative genomic analysis between WHYCs and other YFCs from China. A large number of heritable variants and a suite of promising genes were identified, providing a basis for understanding the adaptive evolutionary history of the breed and its unique traits.

## 2. Materials and methods

### 2.1. Ethics statement

This study was approved by the Animal Ethics Committee of Jiaying University, China. Animal handling and experimentation were conducted according to the animal experimental procedures and guidelines approved by the Animal Ethics Committee of Jiaying University.

### 2.2. Sample collection and sequencing

Wing-vein blood samples were collected from 12 unrelated WHYCs (six males and six females) in Guangdong Kejiahuang Animal Husbandry Co. Ltd, Xingning County, Guangdong Province (24° 9′ 49″ N, 115° 48′ 19″ E). Genomic DNA for each sample was extracted using a standard phenol-chloroform extraction protocol and the DNA libraries were sequenced on the Illumina HiSeq X10 platform (PE150) by Genedenovo Biotechnology Co., Ltd. (Guangzhou, China). Sequencing and base calling were performed following the manufacturer’s protocols. The sequencing data of 12 WHYCs are available in the National Center for Biotechnology Information (NCBI) Sequence Read Archive (SRA) database under accession number PRJNA624239.

For a comparative analysis of the WHYC genomes and those of other YFC breeds, 110 previously published sequences were downloaded from the SRA database (SRP155577). The total average depth across the genomes was 12.65× (S1 Table) [12]. A total of 122 samples were used for the analysis, including 10 Huaibei partridge chickens (HB) from Anhui, 10 Zhengyang Yellow chickens (ZY) from Henan, 10 Jianghan chickens (JH) from Hubei, 10 Hetian chickens (HT) from Fujian, 10 Huanglang chickens (HL) from Hunan, 10 Ningdu Yellow chickens (ND) from Jiangxi, 10 Guangxi Yellow chickens (GX) from Guangxi, 10 Wenchang chickens (WC) from Hainan, and 10 Huiyang bearded chickens (HY), 10 Huaixiang chickens (HX), and 22 WHYC from Guangdong Province, China.

### 2.3. Quality control processing and variant calling

For quality control, the following reads were removed: (1) reads containing more than 10% unidentified nucleotides (N); (2) reads containing more than 50% bases with Phred scores of less than 20; and (3) reads aligned to the barcode adapter. High-quality reads were aligned to the chicken reference genome (Galgal5) [13] assembly using the Burrows-Wheeler Aligner (BWA) [14]. Possible duplicates in the aligned BAM files were sorted and removed utilizing the Picard package’s (picard-tools-1.56) SortSam and MarkDuplicates tools, and local realignment and base quality recalibration were applied using the RealignerTargetCreator, IndelRealigner, and BaseRecalibrator tools from Genome Analysis Toolkit (GATK 2.6–4) [15]. Additionally, sequencing coverage statistics were generated using bedtools (v.2. 25.0) [16].

Variant calling was performed utilizing the GATK Unified Genotyper tool. Single nucleotide polymorphisms (SNPs) and insertion/deletions (InDels) in these 12 chicken genomes were filtered using GATK VariantFiltration, excluding those exhibiting segregation distortion or sequencing errors. Alignment and annotation were performed using ANNOVAR [17]. Structural variants (SVs) were evaluated using the BreakDancer package (Max1.1.2) [18]. Copy number variants (CNVs) were identified using CNVnator (v.0.3.2) [19]. To exclude SNP calling errors caused by incorrect mapping, only high-quality SNPs (filtered by the VariantFiltration of GATK with options "QD < 4.0" -filterName FS -filter "FS > 50.0" -filterName MQ -filter "MQ < 40.0" -G_filterName GQ -G_filter "GQ < 20" -window 15 -cluster 3) were retained for subsequent analyses. Nucleotide diversity (π) was calculated based on SNPs [20].

### 2.4. Selective sweep detection

According to principal components analysis and ADMIXTURE analysis [12], the other 10 YFC breeds were assigned to three groups: (1) south group (SG), including HY, GX, HX, and WC; (2) central group (CG), including HL, ND, HT; and (3) north group (NG), including ZY, JH, and HB. The SNPs within each group were merged. WHYC was treated as the test group, whereas SG, CG, and NG were used as reference groups for comparison.

Evidence for positive selection was investigated in two steps. First, differentiation between the following combinations of populations was evaluated: (1) SG vs. WHYC, (2) CG vs. WHYC, and (3) NG vs. WHYC. The population fixation index (*F*_ST_) [21] and π ratio [22] were estimated for these three comparisons separately. The *F*_ST_ values were calculated with a 100-kb sliding window and 10-kb stepwise increments. The π ratio was determined by calculating the π values of WHYC, SG, CG, and NG using PopGenome [23] in 100-kb windows with 10-kb stepwise increments, and then the ratios (π_SG/πWHYC_, π_CG/πWHYC_, π_NG/πWHYC_) were computed. Allele frequencies at variable sites were used to identify signatures of selection by obtaining outlier values for the π ratio and *F*_ST_. Candidate selective sweeps were chosen in fully overlapping windows with an extremely high π ratio (top 5%) and extremely high *F*_ST_ values (top 5%).

### 2.5. Functional enrichment analysis

The genes in regions with evidence for selection were searched against the Gene Ontology (GO) database (http://www.geneontology.org/) for enrichment analyses of GO terms and Kyoto Encyclopedia of Genes and Genomes (KEGG) pathways. All chicken genes annotated in Ensembl were used as the background set. Q values (false discovery rate) were used for *P*-value correction. Only terms with Q < 0.05 were considered significant.

## 3. Results

### 3.1. Characteristics of the genome datasets

The average genome length was 22,396,626,339 bp after filtering, with a Q30 score of > 94% and GC content of > 44.11% (S2 Table). An average of 164,548,779 clean reads per genome was obtained after strict quality control protocols, including 155,314,465 high-quality clean reads (94.44%). The clean reads were then mapped onto the chicken reference genome (Galgal5) with a mean mapping rate of 85.95%. The average coverage depth was 20.77-fold (ranging from 17.01- to 25.34-fold) for WHYC (Table 1). The average coverage ratio was 97.12% at a sequencing depth target of 1×, 94.83% at 4×, 82.68% at 10×, 37.25% at 20×, and 7.35% at 30× (S3 Table).

**Table 1:** Summary of sequencing data quality of WHYCs.

ID	Clean reads	HQ Clean Reads	Mapped reads	Effective Depth (X)
**A**	184,610,500	177,041,476 (95.90%)	162,606,074 (88.08%)	23.87
**B**	132,229,402	125,886,158 (95.20%)	115,857,466 (87.62%)	17.01
**C**	195,767,428	187,486,964 (95.77%)	172,528,001 (88.13%)	25.34
**H**	154,471,782	146969,390 (95.14%)	133,895,871 (86.68%)	19.66
**E**	145,396,594	132,793,930 (91.33%)	119,761,299 (82.37%)	17.58
**F**	165,678,666	154,833,182 (93.45%)	140,967,959 (85.09%)	20.70
**G**	17,3061,040	161,886,188 (93.54%)	146,834,894 (84.85%)	21.56
**H**	188,061,928	173,728,666 (92.38%)	157,854,546 (83.94%)	23.18
**I**	178,403,862	167,547,588 (93.91%)	149,183,891 (83.62%)	21.91
**J**	143,991,888	135,424,104 (94.05%)	123,521,066 (85.78%)	18.14
**K**	145,924,504	140,743,212 (96.45%)	127,578,971 (87.43%)	18.73
**P**	166,987,750	159,432,728 (95.48%)	146,669,165 (87.83%)	21.53
**Average**	164,548,779	155,314,465 (94.44%)	141,438,267 (85.95%)	20.77

### 3.2. Identification of heritable variation

In total, 11,953,471 SNPs and 1,069,574 InDels (≤ 50 bp) were obtained. SNPs accounted for the majority of variants identified. All genomic variants of the 12 WHYCs in this study are summarized in S1 Fig. The distributions of SNPs and InDels on each chromosome are illustrated in S2A Fig. The number of SNPs and InDels on each chromosome tended to decrease with decreasing chromosome length. Compared with the chicken SNP/InDel database, 1,869,172 (9%) novel SNPs (S2B Fig) and 716,183 (30.28%) novel InDels (S2C Fig) were discovered.

Further annotation of these identified SNPs in the WHYC genome revealed that they are highly enriched in intergenic regions, followed by intronic regions (S3A Fig). Variants in coding regions were mainly nonsynonymous and synonymous SNPs (S3B Fig), including 8,502,106 (71.13%) transitions and 3,451,365 (28.87%) transversions. G-to-A and C-to-T substitutions were the most common transitions at 27%, and A-to-G and T-to-C substitutions accounted for about 22% of the transitions (S3C Fig). The average ratio of transitions to transversions was 2.49. The average numbers of novel transitions and novel transversions per genome were 285,354 and 125,346, respectively. The average ratio of novel transitions to novel transversions was 2.28 (S4 Table). On average, 2,185,071 (40.97%) and 3,148,521 (59.03%) SNPs per genome were homozygous and heterozygous, respectively, 62,657 and 348,043 of which were novel (S5 Table).

The number of individual InDels ranged from 509,979 to 547,304, with an average of 534,391 per genome. Most InDels were located in non-coding regions (S4A Fig). These InDels were mainly frameshift insertions, frameshift deletions, non-frameshift insertions, and non-frameshift deletions (S4B Fig).

In addition to SNPs and InDels, we evaluated SVs in the WHYC genome. The SVs included deletions (DEL), inversions (INV), intra-chromosomal translocations (ITX) and inter-chromosomal translocations (CTX), and their proportions are 18%, 9%, 29%, and 44%, respectively (S5A Fig). CNVs (33,278 in total) were divided into deletions and duplications, revealing a higher percentage of deletions (75.88%) than duplications (24.12%) (S5B Fig). Additionally, 5,782 CNV regions (CNVRs) were obtained, 1,489 (25.75%) of which were shorter than 10 kb and 1,191 (20.60%) were longer than 100 kb (S5C Fig). Among the CNVRs, 3,229 (55.85%) were unique to a single individual, 613 (10.60%) were shared between two individuals, and 1,940 (33.55%) were shared among at least three individuals (S5D Fig).

Compared with other YFC breeds, WHCY had the highest nucleotide diversity (π = 0.0031). We merged the SNPs in the three groups and still detected the highest π value in WHCY among groups (Table 2).

**Table 2:** Nucleotide diversity of 10 chicken breeds analyzed in this study.

Group	SNPs number	Nucleotide diversity (π)	Breed	Nucleotide diversity (π)
**North**	8,621,885	0.0025	HB	0.0027
			ZY	0.0027
			JH	0.0027
**Central**	8,995,103	0.0026	HL	0.0029
			ND	0.0029
			HT	0.0029
**South**	11,274,584	0.0028	HY	0.0029
			HX	0.0029
			GX	0.0029
			WC	0.0029
**WHCY**	11,055,072	0.0031	WH	0.0031

### 3.3. Genome‑wide selective sweep signals

**Fig 1:**
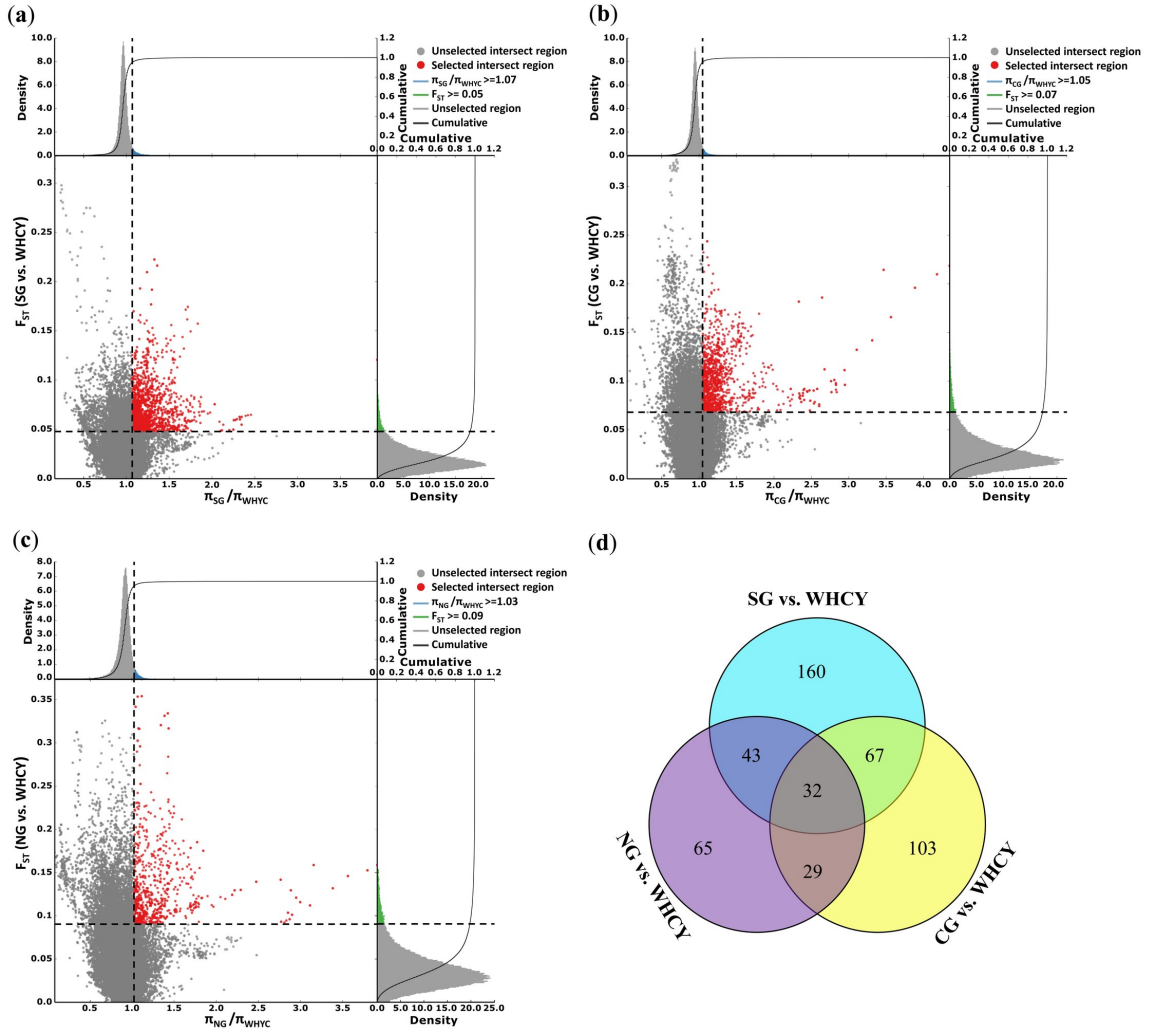
Identification of genomic regions with strong selective sweep signals in Wuhua yellow chicken. Distribution of π radio and *F*_ST_ calculated for 100-kb windows sliding in 10-kb steps. (a) SG vs. WHYC, (b) CG vs. WHYC, and (c) NG vs. WHYC. Red points represent windows fulfilling the selected regions requirement. Genomic regions with both an extremely high π radio (top 5% level) and an extremely high *F*_ST_ value (top 5% level). (d) Venn diagram showing the shared genes between the three comparisons.

**Fig 2:**
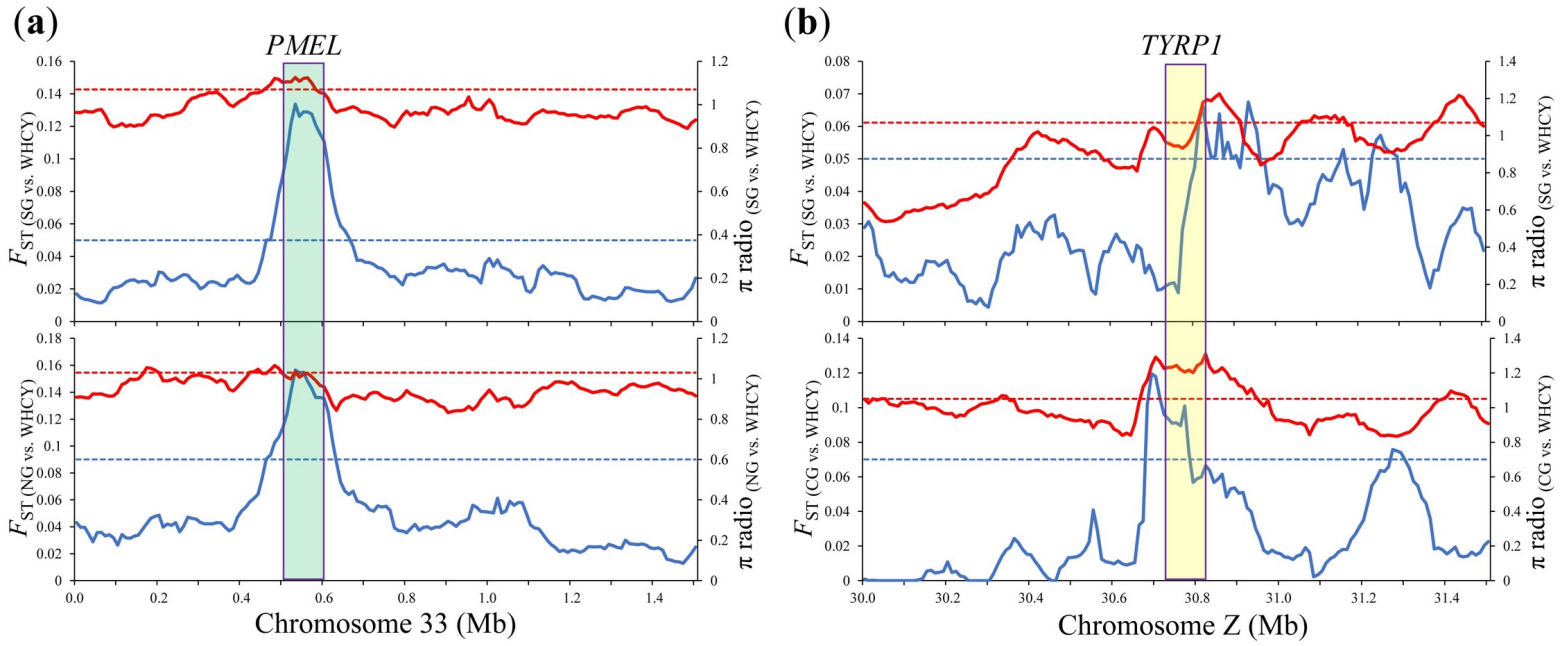
Example of the (a) *PMEL* gene (green box), and (b) *TYRP1* gene (yellow box) with selection signals in Wuhua yellow chicken. *F*_ST_ (blue) and π radio (red), the dotted lines show the threshold *P*-value (0.05).

To detect the signature of selection in WHYC, the 10 YFC breeds mentioned above were classified into three groups (SG, CG, and NG) according to their population structure [12]. Putative regions of selection in the WHYC genome were searched in pairwise comparisons of SG vs. WHYC, CG vs. WHYC, and NG vs. WHYC. Genome-wide screening revealed 302 putative selective sweeps with a π ratio ≥ 1.07 and *F*_ST_ ≥ 0.05 in SG vs. WHYC (Fig 1A and S6A Table), 231 loci with a π ratio ≥ 1.05 and *F*_ST_ ≥ 0.07 in CG vs. WHYC (Fig 2B and S6B Table), and 169 loci with a π ratio ≥ 1.03 and *F*_ST_ ≥ 0.09 in NG vs. WHYC (Fig 1C and S6C Table), spanning 257, 231, and 149 candidate genes, respectively. In addition, 32 loci were shared in the three comparisons, including 31 genes (Fig 1D and S7 Table). In the SG vs. WHYC and NG vs. WHYC comparisons, the *PMEL* gene on chromosome 33, which is associated with feather color, was strongly selected (Fig 2A). In the SG vs. WHYC and CG vs. WHYC comparisons, *TYRP1* on chromosome Z was also strongly selected (Fig 2B). This finding suggested a potential association of these two genes with the distinctive appearance of WHYC among YFC breeds, characterized by white tail feathers and white flight feathers.

### 3.4. GO terms and KEGG pathway enrichment analyses

**Fig 3:**
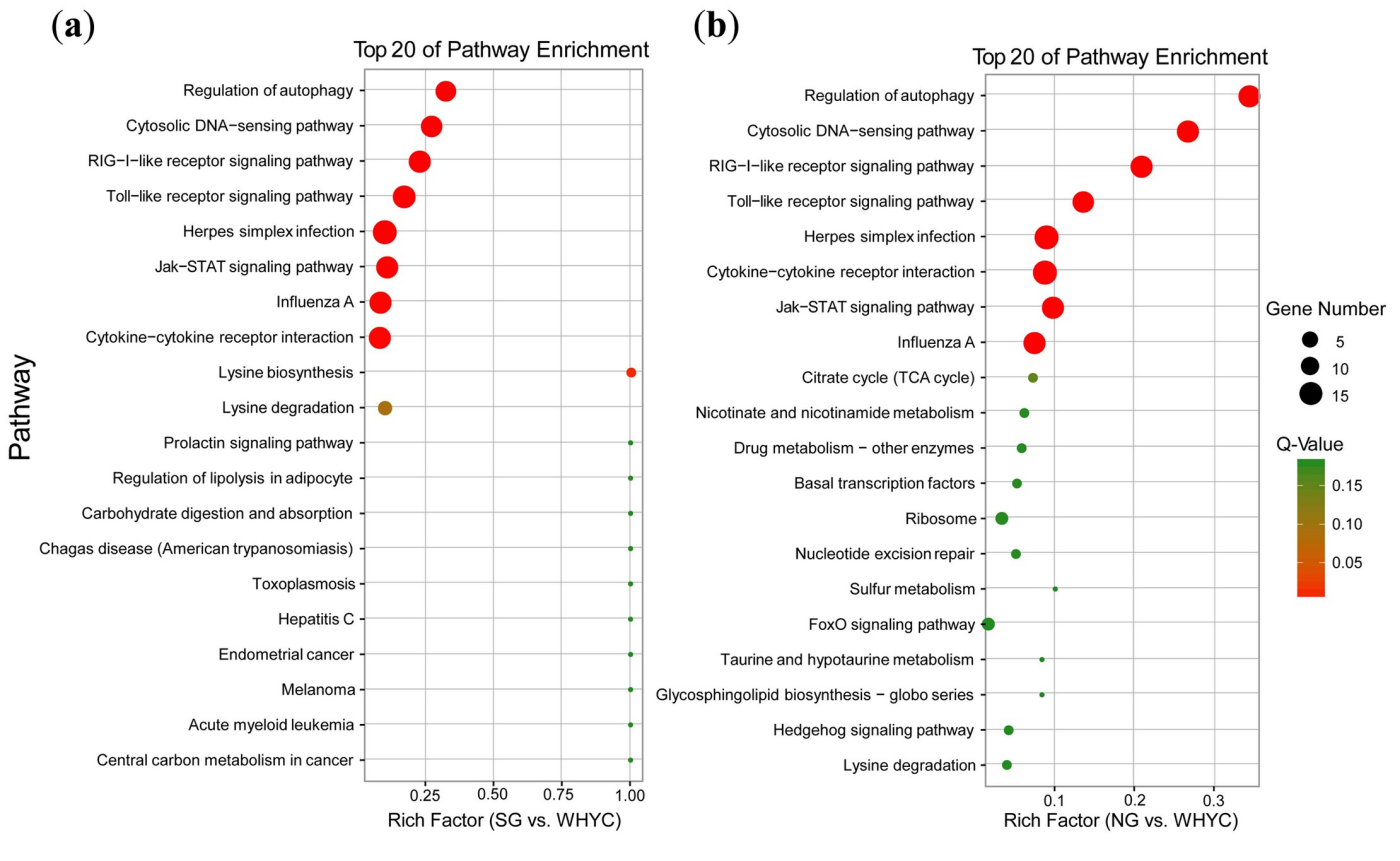
Top 20 of KEGG pathway enrichment analysis of candidate genes under selection in WHYC. (a) SG vs. WHYC, and (b) NG vs. WHYC.

We searched for significantly overrepresented (Q value < 0.05) GO terms and KEGG pathways related to the candidate genes specific to WHYC. One GO term in the molecular function category, lyase activity, was enriched in the CG vs. WHYC comparison. In the KEGG enrichment analysis, eight pathways were identified in the SG vs. WHYC and NG vs. WHYC comparisons, including regulation of autophagy, cytosolic DNA-sensing pathway, RIG-I-like receptor signaling pathway, Toll-like receptor signaling pathway, herpes simplex infection, Jak-STAT signaling pathway, influenza A, and cytokine-cytokine receptor interaction. These pathways involve transport and catabolism, immune system, infectious diseases, signal transduction, and signaling molecules and interaction. In addition, the lysine degradation pathway was enriched at a threshold of *P* ≤ 0.01 in the SG vs. WHYC comparison. Interestingly, most of the enriched clusters were associated with immunity and disease resistance. Many genes were associated with disease resistance, such as *IFNA*, *IFNB*, *ATG10*, *CD86*, *IL11RA*, *VEGFC*, and *IL18* (Table 3 and Fig 3). We conducted GO terms and KEGG pathway enrichment analyses on 32 loci shared by the three comparisons, and only one GO term in the cellular component category (GO:1990391, DNA repair complex) was found to be enriched.

**Table 3:** Gene Ontology (GO) terms and Kyoto Encyclopedia of Genes and Genomes (KEGG) pathways enriched with candidate genes in WHYC.

GO Terms and KEGG Pathways	DEGs ^1^	Genes	*P*-Value	Q-Value	Ref/Test ^2^
GO: 0016829~lyase activity	8	*ADCY10L8*, *ADCY10L3*	0.0002	0.0471	CG/WHYC
ko04140: Regulation of autophagy	13	*IFNA*, *IFNB*	0.0000	0.0000	SG/WHYC
ko04623: Cytosolic DNA-sensing pathway	14	*IFNA*, *IFNB*	0.0000	0.0000	
ko04622: RIG-I-like receptor signaling pathway	15	*IFNA*, *IFNB*, *DDX3X*	0.0000	0.0000	
ko04620: Toll-like receptor signaling pathway	16	*IFNA*, *IFNB*, *CD86*, *PIK3R1*	0.0000	0.0000	
ko05168: Herpes simplex infection	18	*IFNA*, *IFNB*, *UBE2R2*, *CDK2*, *TFIID*, *POLR2A*	0.0000	0.0000	
ko04630: Jak-STAT signaling pathway	15	*IFNA*, *IFNB*, *PIK3R1*	0.0000	0.0000	
ko05164: Influenza A	15	*IFNA*, *IFNB*, *PIK3R1*	0.0000	0.0005	
ko04060: Cytokine-cytokine receptor interaction	15	*IFNA*, *IFNB*, *INHBA*	0.0000	0.0088	
ko00310: Lysine degradation	5	*ALDH7A1*, *AADAT*, *KMT2D*, *COLGALT2*	0.0055	0.0904	
ko04140: Regulation of autophagy	14	*IFNA*, *PRKAA1*, *ATG10*	0.0000	0.0000	NG/WHYC
ko04623: Cytosolic DNA-sensing pathway	14	*IFNA*, *IFNB*, *IL18*	0.0000	0.0000	
ko04622: RIG-I-like receptor signaling pathway	14	*IFNA*, *IFNB*, *MAP3K1*	0.0000	0.0000	
ko04620: Toll-like receptor signaling pathway	13	*MAP3K1*	0.0000	0.0000	
ko05168: Herpes simplex infection	17	*IFNA*, *IFNB*, *UBE2R2*, *SKP2*, *CDK2*	0.0000	0.0000	
ko04060: Cytokine-cytokine receptor interaction	17	*IFNA*, *IFNB*, *IL11RA*, *IL18*, *VEGFC*, *CCL19*	0.0000	0.0000	
ko04630: Jak-STAT signaling pathway	14	*IFNA*, *IFNB*, *IL11RA*	0.0000	0.0000	
ko05164: Influenza A	14	*IFNA*, *IFNB*, *IL18*	0.0000	0.0000	

^1^ differentially expressed genes.

^2^ reference/test group.

## 4. Discussion

We performed whole-genome resequencing of 12 WHYCs to obtain the sequence variants of this breed. A comparative genomic analysis of WHYCs and 10 other YFC breeds (classified into three groups: SG, CG, and NG) revealed signatures of selection, and these genomic regions are potentially associated with disease resistance and the white feather trait. These results lay a solid foundation for utilizing the valuable genetic resources of WHYCs.

SNPs account for about 90% of all genetic variants [24], and are widely used in genetic research owing to the high density, low cost, and applications to large-scale population testing [25]. In this study, 11,953,471 SNPs in the WHYC genome were identified, exceeding estimates in the Silkie (5,385,458, 23-fold) and Taiwan country chicken L2 (5,142,622, 25-fold) breeds [26]. Compared with other YFCs, nucleotide diversity was the highest in WHYC, suggesting that this breed maintains substantial variation and is therefore a valuable genetic resource. After SNPs, InDels were the most abundant mutation type in the genome. Chicken feather color [27] and the creeper trait [28] are associated with InDels. A total of 1,095,574 InDels were detected in this study, which is fewer than the estimate obtained by Yan [29], who studied 12 chicken breeds (seven Chinese indigenous breeds, four commercial breeds, and one red jungle fowl), compared with our study that included 12 individuals of a single breed. Additionally, the higher percentage of novel InDels (30.28%) than novel SNPs (9%) in the chicken SNP/InDel database indicates that InDels in the chicken genome are not sufficiently characterized.

SVs are major sources of genetic variation and may account for a substantial portion of the missing heritability in population genetic studies [30]. SVs can give rise to new genes [31] and contribute substantially to both disease susceptibility/resistance and general phenotypic variation in chickens [32, 33]. For instance, the chicken pea-comb phenotype is associated with a CNV in intron 1 of SOX5 [34], dermal hyperpigmentation is associated with rearrangement of the EDN3 locus [35], and late feathering is associated with a partial duplication of PRLR [36]. In addition, the chicken comb [37] and beard [38] traits are associated with SVs. Therefore, the 41,408 SVs (including 33,278 CNVs) identified in this study can be used to identify additional resistance-related loci in WHYC.

Disease resistance is an important trait in poultry, directly affecting mortality, growth rate, and production performance in poultry farming [39]. In a KEGG enrichment analysis, five pathways related to the immune system or infectious diseases were significantly enriched, including the cytosolic DNA-sensing pathway, RIG-I-like receptor signaling pathway, Toll-like receptor signaling pathway, herpes simplex infection, and influenza A. The cytosolic DNA-sensing pathway involves specific families of pattern recognition receptors that detect and generate an innate immune response when foreign DNA invades the host cell [40, 41]. The RIG-I-like receptor signaling pathway is an important part of the innate response to viral infections, which is jointly regulated by stimulation and inhibition signals to promote virus clearance and reduce immune-mediated pathology [42]. The Toll-like receptor family members recognize conserved microbial structures such as viral double-stranded RNA and bacterial lipopolysaccharides. Moreover, they can activate signaling pathways, leading to immune responses against microbial infections [43]. The lysine degradation pathway was enriched in the SG vs. WHYC comparison. When available carbohydrates are insufficient, lysine is involved in ketone production and glucose metabolism [44]. Lysine can also regulate the functions of the thymus and spleen via neuroregulatory channels, thereby improving anti-stress activity and immunity [45].

In chickens, breed traits are linked to genetic variation [46–48]. We detected variation in several genes related to disease resistance. For example, inhibin subunit beta A (*IFNA*) encodes interferon alpha and interferon omega 1 (*IFNB*) encodes interferon beta. Interferons confer anti-virus and anti-tumor immunity. They can activate natural killer cells to kill cells infected by viruses and can induce the expression of major histocompatibility complex I [49]. CD 86 molecule (*CD86*) encodes a type I membrane protein belonging to the immunoglobulin superfamily that is involved in the regulation of T cell activation [50]. When stimulated by inflammation, the upregulation of CD86 expression in dendritic cells overrides the immunosuppressive function, leading to immune activation [51]. Interleukin 18 (*IL-18*) encodes a proinflammatory cytokine that enhances the natural killer cell activity of spleen cells and stimulates T-helper type I cells to produce interferon. Degen et al. [52] reported that rHis-ChIL-18 augments the antibody response to Clostridium perfringens α-toxoid and Newcastle disease virus antigens. Additionally, the protective efficacy of the rFPV-HA vaccine can be significantly enhanced by *IL-18* [53]. Accordingly, it is a safe immunostimulator in chickens. *IL11RA* encodes the IL-11 receptor, and mutations in this gene cause autosomal recessive Crouzon‐like craniosynostosis [54] and affect thymus immune function [55]. Vascular endothelial growth factor C (*VEGFC*) is a determinant of lymphatic vessel density, tumor staging, and lymph node metastasis, and is associated with the failure of nasopharyngeal carcinoma to respond to radiotherapy [56]. Autophagy related 10 (*ATG10*) is a critical gene for autophagy and cancer, and there is increasing evidence for the importance of autophagy-related genes in the maintenance, therapy, and pathogenesis of cancer [57]. In colorectal cancer, increased *ATG10* expression is associated with lymph node metastasis and lymphovascular invasion [58]. *ATG10* is a target gene of miR-369-3p, which inhibits cell proliferation and migration by targeting cancer cells via autophagy in endometrioid adenocarcinoma [59].

Chinese indigenous chickens often exhibit strong resistance to disease. In recent decades, gene introgression from commercial lines to various Chinese indigenous chickens has been observed [60]. This process will continually reduce breed specificity, which is a particular issue for breeds with distinct characteristics [61]. Nevertheless, adverse geographical or economic conditions protect against introgression [60]. Compared with other YFCs, the WHYC production region is in a remote mountainous area with a relatively harsh environment and poor economic conditions, providing a barrier to gene introgression from commercial lines and enabling the maintenance of strong disease resistance. In addition, WHYC has been exported to other regions but has never been used for large-scale breeding, only existing as a free-range model. Therefore, vaccination has rarely been used in the breeding process. The breed relies exclusively on autoimmunity for resistance to various diseases, which can explain the strong disease resistance. Based on these characteristics, it is worthwhile to attempt to breed WHYC into a typical disease-resistant chicken such as Fayoumi chicken, which can further be applied to broader chicken breeding.

Feather color is an important visual characteristic of chickens. The species is rich in feather polymorphisms, including breeds with different feather colors. Notably, WHYC is the only traditional YFC breed with white tail feathers and white flight feathers. To date, *MC1R* [62], *PMEL* [63], *CDKN2A* [64], *SLC45A2* [65], *SOX10* [27], and *TYR* [66] variants have been reported to be responsible for or associated with feather color. *PMEL* is an important candidate gene affecting feather color that plays a key role in the early development of eumelanosomes from nearly spherical to elliptical [67]. In chickens, *PMEL* gene polymorphisms are associated with the Dominant white, Dun, and Smoky color variants [63] *TYRP1*, a member of the *TYR* gene family, encodes a melanosomal enzyme and plays a critical role in the melanin biosynthetic pathway [68]. This gene can affect plumage color in poultry. For example, the chocolate plumage color in chickens is associated with a missense mutation in *TYRP1* [69]. In this study, we found that *PMEL* and *TYRP1* are strongly selected in WHYC. We speculate that the white tail feather and white flight feather traits of WHYC may be linked to these two genes, but further experimental verification is needed.

Meat quality is another important aspect of chickens. However, we did not detect enrichment for genes associated with meat quality. The most plausible explanation is that the analysis only included Chinese indigenous YFC populations, which are renowned for their good meat quality [12].

## 5. Conclusions

In summary, a comprehensive whole-genome map of WHYC was generated and heritable variation was characterized. Moreover, several pathways and genes related to the immune system and infectious diseases were detected, proving an insight into the molecular mechanisms underlying the strong disease resistance of WHYC. Additionally, *PMEL* and *TYRP1*, associated with the regulation of feather color, were found to be under selection in this breed. These findings provide a foundation for future studies on the molecular basis of phenotypic variation and disease in WHYC and other chickens and will facilitate the understanding of the germplasm characteristics and utilization potential of this breed.

## Supporting information

S1 Fig Summary of genomic variant landscape per chromosome of all 12 chicken genomes sequenced in this study. Circos plot of genome variants. Different loops from outside to inside summarize the length of each chromosome (unit: Mb), gene density, SNP density (SNP density > 0.0015 is marked by a red square, 0.0005 < SNP density ≤ 0.0015 by a gray circle, and SNP density ≤ 0.0005 by a green triangle), positions of INS (structural variation of the insertion type), positions of INV (structural variation of the inverted type) on the chromosome, and the positions of ITX (structural variation of the intrachromosomal translocation type). The lines of the inner circle indicate positions of CTX (structural variation of the interchromosomal translocation type) on chromosomes.
(TIF)

S2 Fig Distribution of Wuhua yellow chicken’s SNPs and InDels. (a) The number of SNPs and InDels on each chromosome; (b) The SNPs map to SNP database; (c) The InDels map to InDel database.
(TIF)

S3 Fig Annotation and transition-transversion analysis of the clean genomic SNPs of all 12 chickens sequenced in this study. Proportions of SNPs are classified according to: (a) the genomic locations in which they occur, (b) The genetic coding attributes, and (c) bars represent the total number of transitional SNPs (red) followed by the individual base transitions types, and the total number of transversion SNPs (green) followed by the individual base transversion types. Numbers and proportions of the two groups of variants are their constituent variants are shown.
(TIF)

S4 Fig Annotation of the clean InDels of the 12 chicken genomes generated in this study. The proportion of InDels classified according to: (a) the genomic locations in which they occur, and (b) genetic coding attributes.
(TIF)

S5 Fig Summary of the structural variations (SVs) in the 12 chicken genomes generated in this study. (a) Proportion of different SV types. CTX (interchromosomal translocation), DEL (deletions), INV (inversion), ITX (intrachromosomal translocation); (b) Number of CNVs of deletion and duplication types identified in the WHYC genomic dataset; (c) The length distribution of CNVRs; (d) The frequency distribution of CNVRs.
(TIF)

S1 Table Genomic references used for comparison in this study. (XLSX)

S2 Table Base information statistics table before and after quality filtering of the chicken sequenced in this study. (XLSX)

S3 Table Summary of the average sequencing coverage of all 12 chicken genomes generated in this study. (XLSX)

S4 Table Individual transition and transversion SNPs statistics of the 12 chickens sequenced in this study. (XLSX)

S5 Table Annotation of the hybrid status of SNPs in each chicken genome sequenced in this study. (XLSX)

S6 Table The putative locus and candidate genes of WHYC. (XLSX)

S7 Table 32 loci were shared in the three groups. (XLSX)
