# Supplementary figures and images for: Genomic variations and signatures of selection in Wuhua yellow chicken
Source: PLoS One. 2020 Oct 23;15(10):e0241137. doi: 10.1371/journal.pone.0241137 (PMC7584229; doi:10.1371/journal.pone.0241137)

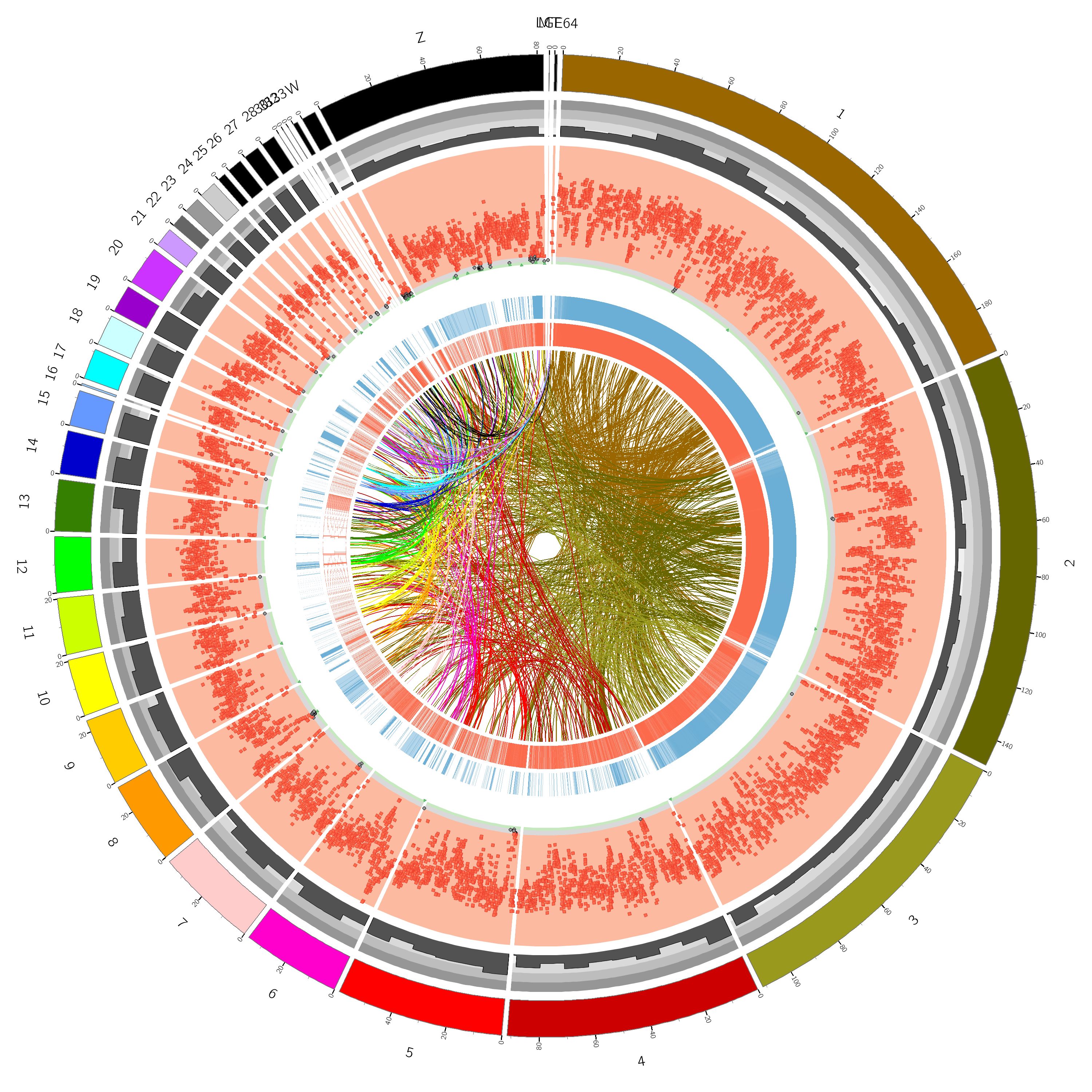

Supplement: S1 Fig — Circos plot of genome variants. Different loops from outside to inside summarize the length of each chromosome (unit: Mb), gene density, SNP density (SNP density > 0.0015 is marked by a red square, 0.0005 < SNP density ≤ 0.0015 by a gray circle, and SNP density ≤ 0.0005 by a green triangle), positions of INS (structural variation of the insertion type), positions of INV (structural variation of the inverted type) on the chromosome, and the positions of ITX (structural variation of the intrachromosomal translocation type). The lines of the inner circle indicate positions of CTX (structural variation of the interchromosomal translocation type) on chromosomes. (TIF) [file pone.0241137.s001.tif]

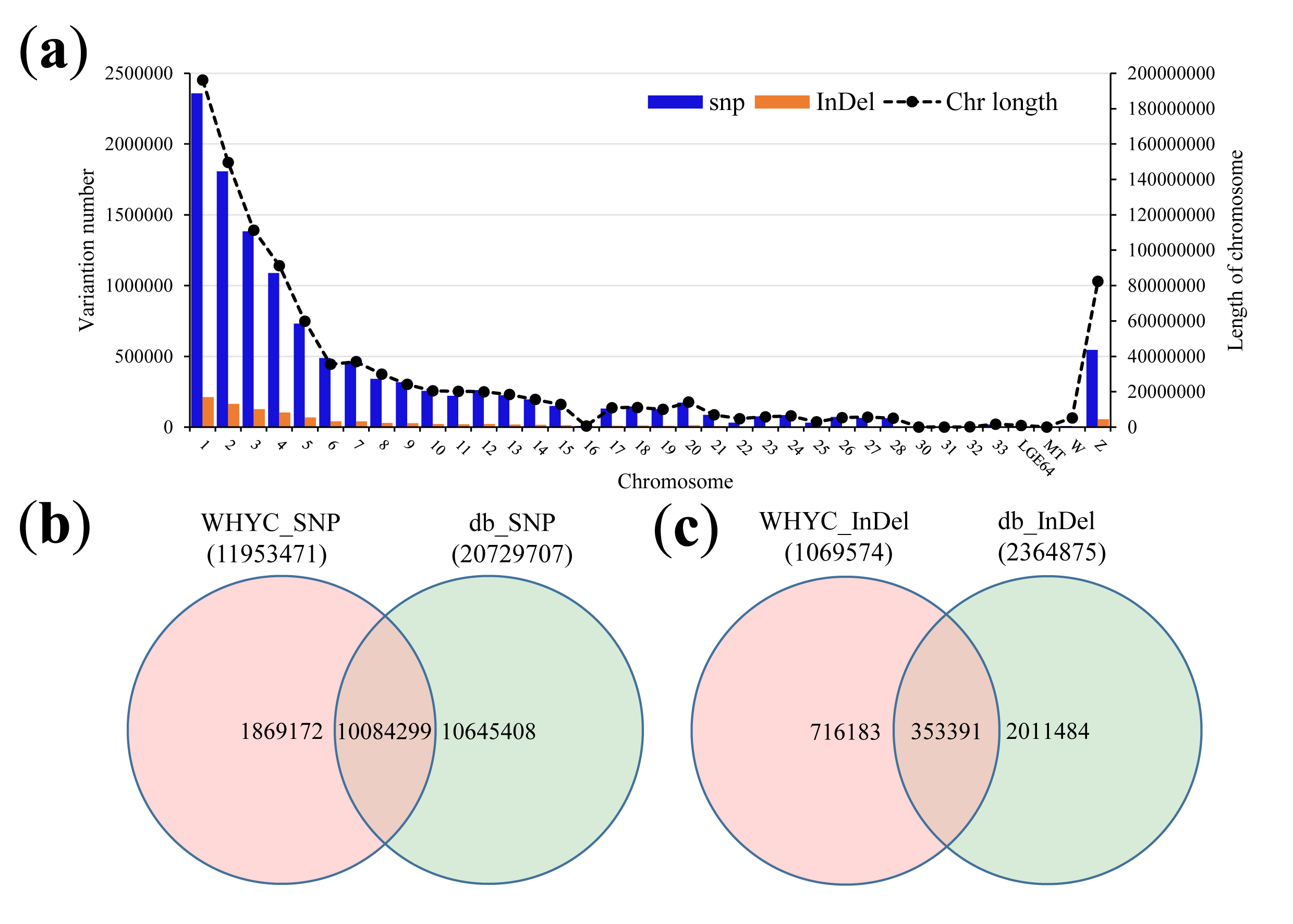

Supplement: S2 Fig — (a) The number of SNPs and InDels on each chromosome; (b) The SNPs map to SNP database; (c) The InDels map to InDel database. (TIF) [file pone.0241137.s002.tif]

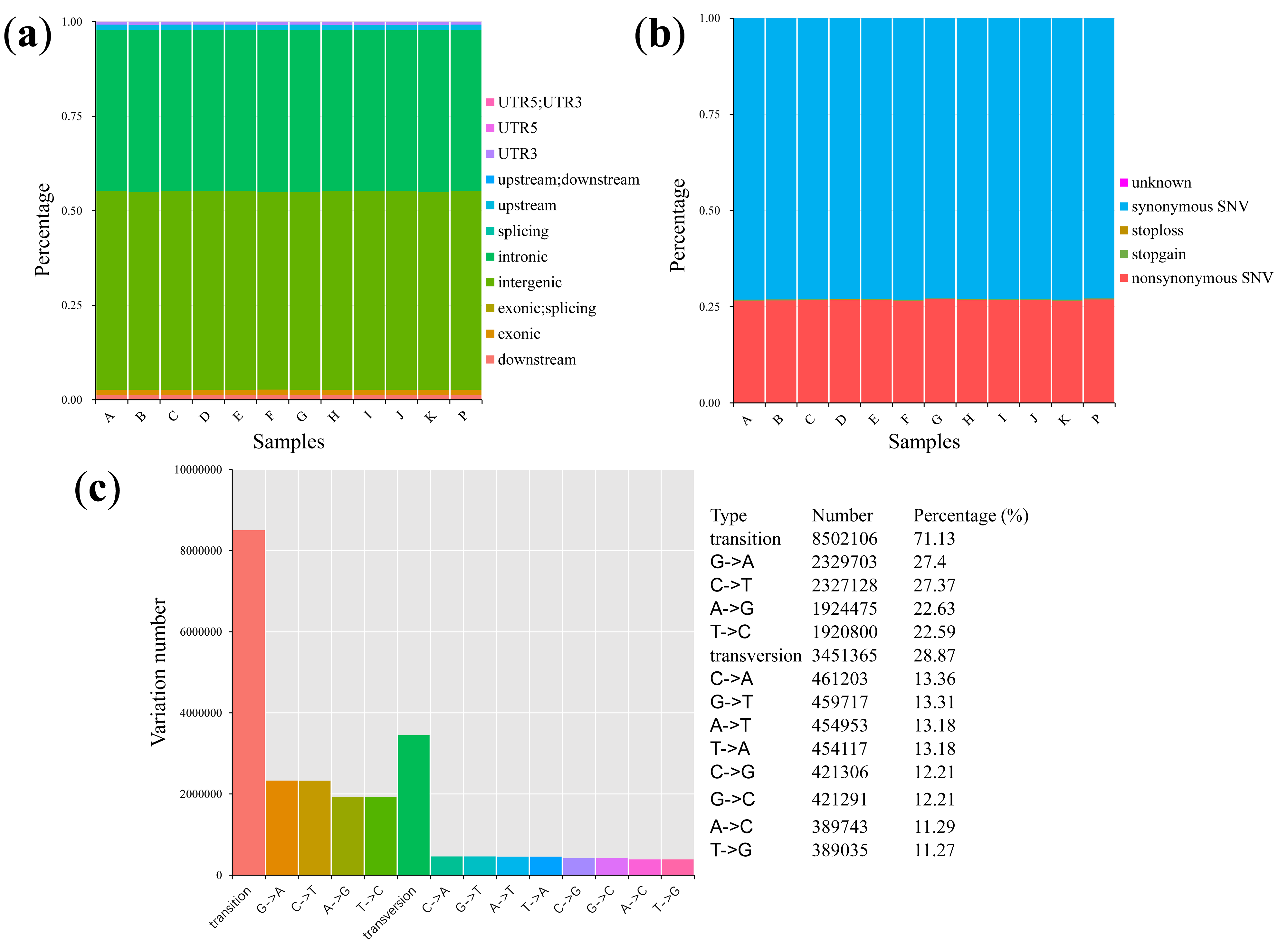

Supplement: S3 Fig — Proportions of SNPs are classified according to: (a) the genomic locations in which they occur, (b) The genetic coding attributes, and (c) bars represent the total number of transitional SNPs (red) followed by the individual base transitions types, and the total number of transversion SNPs (green) followed by the individual base transversion types. Numbers and proportions of the two groups of variants are their constituent variants are shown. (TIF) [file pone.0241137.s003.tif]

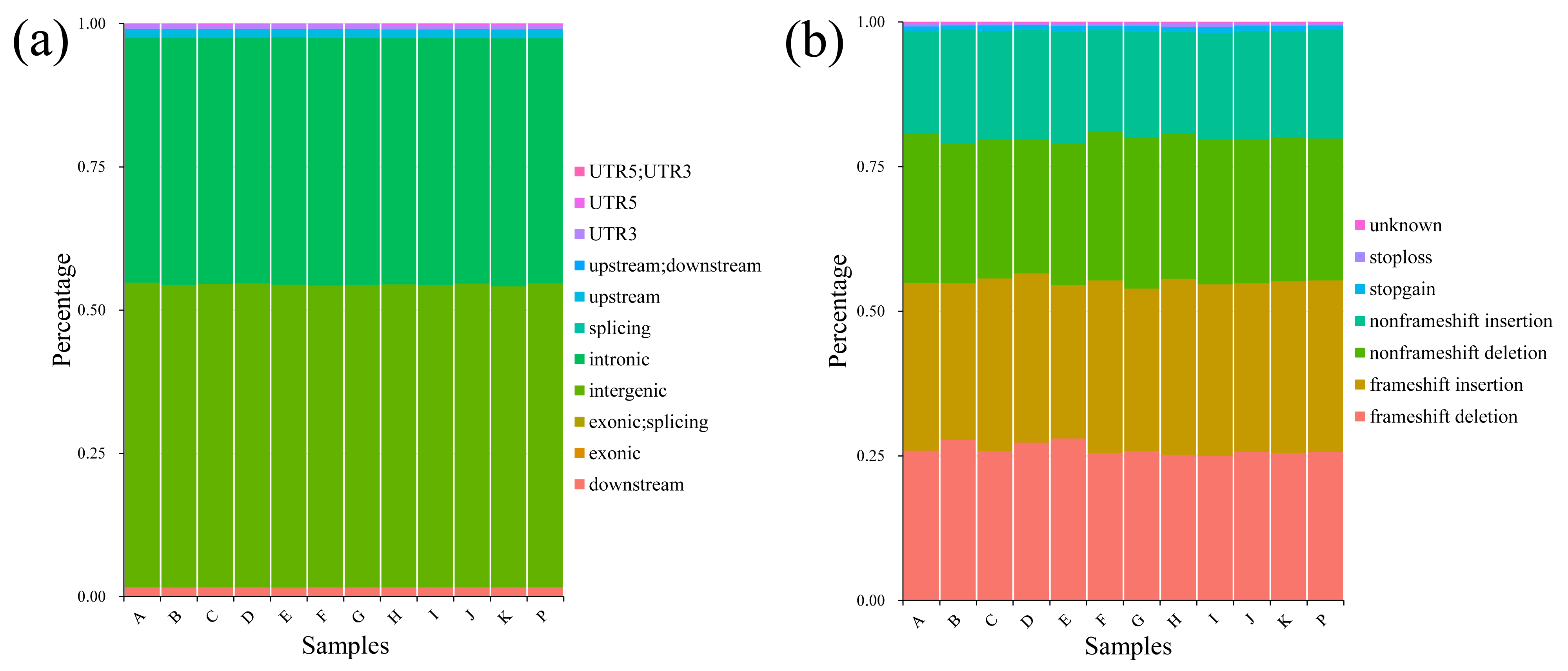

Supplement: S4 Fig — The proportion of InDels classified according to: (a) the genomic locations in which they occur, and (b) genetic coding attributes. (TIF) [file pone.0241137.s004.tif]

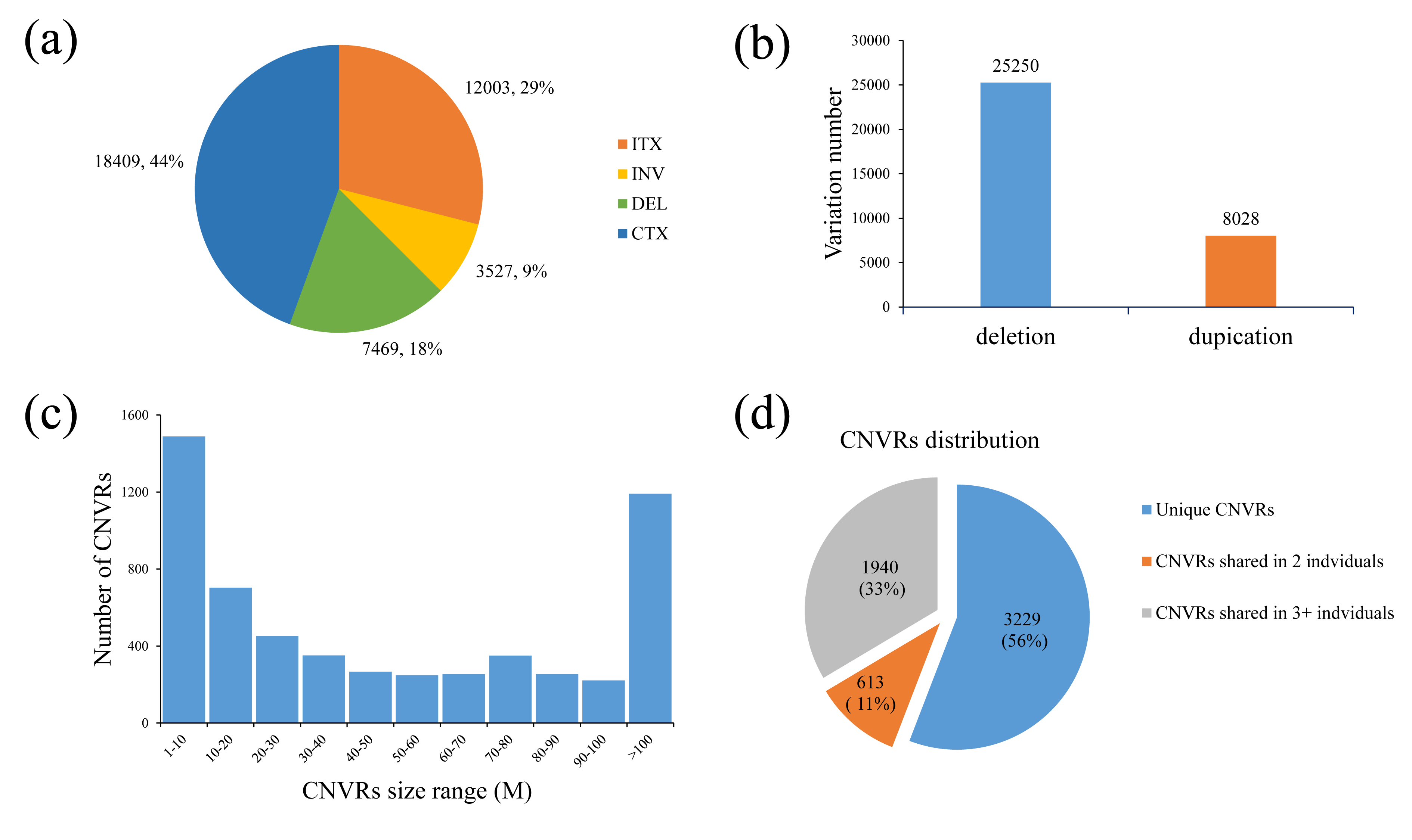

Supplement: S5 Fig — (a) Proportion of different SV types. CTX (interchromosomal translocation), DEL (deletions), INV (inversion), ITX (intrachromosomal translocation); (b) Number of CNVs of deletion and duplication types identified in the WHYC genomic dataset; (c) The length distribution of CNVRs; (d) The frequency distribution of CNVRs. (TIF) [file pone.0241137.s005.tif]
